# Structural and Connectivity Alterations of the Premotor Cortex in Autistic Children: Implications for Affective Motor Impairments

**DOI:** 10.3390/brainsci16050446

**Published:** 2026-04-23

**Authors:** Cecilia Carapelli, Marzio Gerbella, Francesca Tambuscio, Giuseppe Di Cesare

**Affiliations:** 1 Department of Food and Drugs, University of Parma, 43121 Parma, Italy; cecilia.carapelli@unipr.it; 2 Department of Medicine and Surgery, University of Parma, 43125 Parma, Italy; marzio.gerbella@unipr.it (M.G.); francesca.tambuscio@unipr.it (F.T.); 3 Cognitive Architecture for Collaborative Technologies Unit, Italian Institute of Technology, 16152 Genova, Italy

**Keywords:** autism spectrum disorder, vitality forms, premotor cortex, white-matter connectivity, voxel-based morphometry, probabilistic tractography, social communication

## Abstract

**Highlights:**

**What are the main findings?**

- Children with autism spectrum disorder (ASD) showed increased grey-matter volume in the premotor cortex (PM), dorsolateral prefrontal cortex (DLPFC), and middle cingulate cortex (MCC), with no differences in the dorso-central insula (DCI).
- Diffusion imaging revealed white-matter microstructural alterations and atypical tract organization in premotor-related pathways connecting the PM with the DLPFC, MCC, and DCI in ASD.

**What are the implications of the main findings?**

- The results indicate atypical structural and connectivity development within the Vitality Forms network in children with ASD.
- These neural alterations may underlie differences in processing affective components of action during social interactions in ASD.

**Abstract:**

When people interact, their actions reflect mood, attitude, and intention. Stern termed the affective qualities conveyed by actions, such as gentleness or rudeness, Vitality Forms (VFs). Previous research shows that children with autism spectrum disorder (ASD) differ from neurotypical (NT) peers in both perceiving and expressing these fundamental aspects of communication. It remains unclear whether these differences arise from structural or connectivity alterations in brain regions involved in VF processing. This study investigated structural and microstructural brain differences between children with ASD and NT peers, focusing on the VF-related network, which includes the dorso-central insula (DCI), premotor cortex (PM), middle cingulate cortex (MCC), and dorsolateral prefrontal cortex (DLPFC). Structural MRI data were collected from 60 right-handed boys aged 6–10 years (30 ASD, 30 NT), with diffusion MRI data available for a subset (20 ASD, 20 NT). A multimodal approach combined voxel-based morphometry (VBM), tract-based spatial statistics (TBSS), and probabilistic tractography. VBM revealed increased grey-matter volume in the PM, DLPFC, and MCC in the ASD group, with no differences in the DCI. TBSS showed white-matter microstructural alterations in premotor-related pathways. Probabilistic tractography further indicated atypical organization of tracts connecting the PM with the DLPFC, MCC, and DCI in children with ASD. Overall, the findings suggest atypical development of the premotor cortex and its associated white-matter connections in ASD, supporting theoretical accounts that link this network to altered processing of affective action dynamics during social interaction.

## 1. Introduction

In the last few years, increasing attention has been paid to the neural basis of action vitality forms (VFs) [1,2], which represent the affective component of an action, specified in terms of its dynamics and kinematics. VFs are a fundamental aspect of social communication because they allow people, on the one hand, to convey their own mood or attitude and, on the other, to perceive and understand the mood or attitude of others. Several functional magnetic resonance imaging (fMRI) studies have identified a brain network involved in the expression and recognition of hand-related action VFs [3,4]. Specifically, this network includes the dorso-central insula (DCI), the premotor cortex (PM), the dorsolateral prefrontal cortex (DLPFC), and the middle cingulate cortex (MCC). Recent findings suggest that during the observation of action VFs, the DCI modulates the activity of PM, whereas during action execution the PM modulates DCI activity. These reciprocal modulatory effects, occurring during the encoding of VFs, are thought to be critical for transforming the affective component of an observed action into specific kinematic features that could then be reused during the execution of a subsequent action, thereby ensuring fine tuning between two interacting individuals [5]. The possibility of exchanging information among these regions is corroborated by tractography findings demonstrating anatomical connections among them in both humans and monkeys [6]. If the processing of VFs is related to the functioning and mutual exchange of information within this neural pathway (including PM, DLPFC, DCI, MCC), an important open issue is whether impairments in VF processing, such as those observed in children with autism, may be associated with alterations in the structural organization or connectivity of this network. Previous findings show that children with autism spectrum disorder (ASD) can recognize [7,8] and express [9] VFs, but in a different way from neurotypicals (NTs). Furthermore, these studies proposed that the understanding and expression of VFs in individuals with ASD predominantly rely on higher-order cognitive strategies rather than on the automatic motor resonance mechanisms that characterize NT children. However, the neuroanatomical architecture potentially supporting these atypical processing patterns remains largely unexplored.

The present study addresses this gap by examining whether impairments in VF processing in ASD may be related to alterations in the structural and microstructural organization of the VF-related brain network. To this purpose, we employed a multimodal neuroimaging approach combining voxel-based morphometry (VBM), tract-based spatial statistics (TBSS), and probabilistic tractography. This integrative methodology allowed us to assess both grey matter morphology and white matter connectivity within the VF network in children with ASD compared to neurotypical controls.

## 2. Methods

### 2.1. Participants

Participants were selected from the Autism Brain Imaging Data Exchange (ABIDE), a multisite repository of neuroimaging and phenotypic data https://fcon_1000.projects.nitrc.org/indi/abide/ (accessed on 19 April 2026) [10,11]. Data were contributed by three sites: NYU Langone Medical Center, San Diego State University, and Trinity Centre for Health Sciences. Consistent with our previous studies [8,12], we included only right-handed male children aged 6–10 years with a full-scale IQ above 70, as reported in the ABIDE phenotypic dataset. The decision to focus exclusively on male children stems from the distinct symptomatic manifestations observed between the two sexes [13,14]. Furthermore, this approach ensures continuity with our previous research, which was conducted on exclusively male cohorts [7,8,9]. The final structural MRI sample comprised 60 participants with usable T1-weighted images (30 ASD: 8.74 ± 1.58 years; 30 NT: 8.50 ± 1.26 years). Diffusion-weighted MRI data were available for a subset of 40 participants (20 ASD: 8.82 ± 1.47 years; 20 NT: 8.76 ± 1.15 years). Acquisition parameters and diffusion acquisition details are reported in Supplementary Tables S1 and S2. For the ASD group, we additionally extracted clinical severity measures from the ABIDE phenotypic files, including Autism Diagnostic Interview-Revised (ADI-R) scores [15,16,17], focusing on the Reciprocal Social Interaction (RSI) score, and Autism Diagnostic Observation Schedule, Second Edition (ADOS-2) scores [18], focusing on Social Affect (SA) and calibrated severity metrics [19]. Demographic and clinical characteristics of the structural sample and diffusion subset are summarized in Table 1. Exclusion criteria for the ASD group included an ADOS-2 total score below 10. This threshold was applied to ensure that participants met a clinically meaningful level of autism severity, consistent with our previous studies (see above). All data were anonymized and publicly released by the ABIDE consortium.

### 2.2. Data Processing and Neuroimaging Approach

To investigate group differences in brain structure and connectivity between ASD and NT children, we implemented three complementary neuroimaging approaches: (1) VBM approach to assess regional grey-matter morphology; (2) TBSS approach to examine white-matter microstructural measures on a common skeleton; and (3) probabilistic tractography to reconstruct structural pathways within an a priori defined VF network.

### 2.3. Voxel-Based Morphometry (VBM)

Voxel-based morphometry was performed in SPM12 (Wellcome Centre for Human Neuroimaging, UCL) running in MATLAB (v2025b). Tissue segmentation used the unified segmentation framework with bias-field correction and tissue classification into grey matter (GM), white matter (WM), and cerebrospinal fluid (CSF) [20,21]. Given the pediatric age range, age-adjusted tissue probability maps (TPMs) were generated using the Template-O-Matic toolbox [22] and used as priors in the segmentation step. To improve inter-subject registration, a study-specific template was created from individual tissue class images using diffeomorphic registration (geodesic shooting) [23]. GM images were then normalized to MNI space via the study-specific template and modulated by the Jacobian determinants to preserve local volumetric information. Modulated GM maps were smoothed with a Gaussian kernel of 4 mm FWHM. Total intracranial volume (TIV) was estimated as the sum of native-space GM, WM and CSF volumes obtained from SPM outputs. Second-level statistics were conducted using a two-sample *t*-test comparing ASD and NT groups. To address key confounds inherent to multisite ABIDE data, site/scanner was modelled as a nuisance factor using dummy-coded regressors (one per site, with one site serving as reference), age, full-scale IQ and TIV were included as covariates of no interest. The distribution of ASD and NT participants was comparable across sites, as indicated by similar site-wise percentages in each group (see Table 1), thereby reducing the risk of group-by-site collinearity. Statistical inference was restricted to an a priori mask encompassing the VF network (see below) and assessed using small-volume correction within the explicit mask (voxel-wise p_FWE < 0.05, cluster-forming threshold *p* < 0.001 uncorrected).

### 2.4. A Priori VF Network Mask (Explicit ROI Mask)

The explicit VF network mask comprised spherical ROIs (radius 5 mm) centred on published activation peaks associated with VF processing in the DCI, PM, MCC, and DLPFC [4,5]. Coordinates (MNI space) and ROI definitions are reported in Supplementary Table S3. Spheres were created in MNI space and combined into a single binary mask; the final mask was resliced to match the voxel size and dimensions of the normalized GM maps used in group analyses.

### 2.5. Tract-Based Spatial Statistics (TBSS)

Diffusion-weighted images were brain-extracted from the b0 image and corrected for eddy-current-induced distortions and head motion using an eddy-current/motion correction framework with outlier detection and replacement when available [24,25]. Data quality was assessed via visual inspection of corrected diffusion images and review of eddy-derived QC outputs (e.g., outlier slices/volumes and motion estimates). All diffusion datasets included in the final sample passed preprocessing and quality control, and no additional participants were excluded at this stage. The reduced MRI sample size reflects data availability within the ABIDE database rather than post hoc exclusion. A diffusion tensor model was fitted to the corrected data to derive fractional anisotropy (FA), mean diffusivity (MD), and eigenvalue maps (L1, principal value; L2, L3, secondary values). Axial diffusivity (AD) was defined as L1, and radial diffusivity (RD) was computed as (L2 + L3)/2. FA maps were processed using the standard TBSS pipeline [26]. Given the pediatric age range, nonlinear registration used a study-representative target strategy, and the mean FA image and skeleton were derived from the study sample to optimize alignment in a young cohort. The mean FA skeleton was thresholded at FA > 0.20, and each participant’s FA data were projected onto the skeleton. Non-FA tensor-derived measures (MD, AD, RD) were subsequently aligned and projected onto the same skeleton using the standard TBSS non-FA approach. Considering that FA changes in regions with crossing fibres can be difficult to interpret, we additionally estimated fibre-specific measures using Bayesian multi-fibre modelling [27,28]. Specifically, a two-fibre-per-voxel model was fitted to obtain fibre orientation distributions and partial volume fractions for the first and second fibre populations (F1 and F2). To ensure that a given fibre population corresponded consistently across participants, i.e., to mitigate orientation “swapping” of the first and second fibre compartments between subjects—fibre-specific TBSS processing followed the established crossing-fibres TBSS framework [29]. Skeletonized F1 and F2 maps were then generated and analyzed as complementary endpoints to increase interpretability in crossing-fibre regions.

### 2.6. VF Network White-Matter ROI Mask (Skeleton-Constrained)

To focus inference on white matter anatomically related to VF network nodes and to avoid whole-skeleton testing, voxel-wise TBSS were restricted to an a priori defined VF-network white-matter ROI mask. Starting from the cortical VF network ROIs used in the VBM analysis (5 mm spheres centred on a priori MNI coordinates, see Supplementary Table S3) corresponding white-matter ROIs were defined in the adjacent subcortical white matter beneath each cortical node on the group mean FA image, following predefined anatomical criteria. These white-matter ROIs were constrained to voxels belonging to the TBSS skeleton (FA > 0.20). All ROIs were binarized and resampled into the TBSS skeleton space using nearest-neighbour interpolation to preserve label integrity. The resulting white-matter ROIs were combined into a single binary VF-network mask, which was used to constrain voxel-wise TBSS statistical analyses. ROI masks were finalized prior to running group-level TBSS comparisons.

### 2.7. Statistical Inference

Voxel-wise group comparisons were performed using permutation-based nonparametric testing with threshold-free cluster enhancement (TFCE) and (FWE) correction [30,31]. Statistical significance was set at p_FWE < 0.05 within the VF-network TBSS mask. The design matrix included Group (ASD vs. NT) and nuisance covariates accounting for multisite ABIDE confounds such as the site/scanner, age, full-scale IQ, and a diffusion motion index derived from eddy QC outputs (e.g., mean volume-to-volume displacement). FA was treated as the primary diffusion endpoint; MD, AD, RD, L2–L3, and fibre-specific measures (F1, F2) were analyzed as complementary measures using the same inferential framework. The number of permutations was set to 5000.

### 2.8. Correlation Analysis

To explore associations between white-matter microstructural alterations and clinical severity, post hoc correlations were performed within the ASD subgroup for diffusion metrics showing significant group effects in the TBSS analysis.

### 2.9. Probabilistic Tractography

Probabilistic tractography was used to characterize structural connectivity within an a priori defined VF network, previously identified in functional and anatomical studies. Based on this network definition, three ipsilateral pathways in the left hemisphere were reconstructed: PM-DCI, PM–MCC, and PM–DLPFC. Diffusion data were modelled using a Bayesian crossing-fibre approach, and tractography was performed in each participant’s native diffusion space to minimize registration bias in a pediatric cohort. The centres of the seed and target regions of interest were defined in native diffusion space according to detailed anatomical criteria. Then, they were transformed into spherical ROIs (5 mm radius), binarized, and used for probabilistic tracking. For each ROI pair, tracking was run bidirectionally and the resulting connectivity distributions were combined to obtain a single tract representation. Individual tract density maps were thresholded at 10% of the subject-specific maximum connectivity value to reduce spurious streamlines and then binarized. Group overlap maps were generated by summing binarized tracts across participants. Tract reconstruction rate was defined as the percentage of participants showing a non-empty suprathreshold tract map. In addition, for each pathway, a tractography-derived connectivity index (CI) was calculated from the two directional tracking runs. For each direction, a normalized connectivity value was computed as the ratio between way-total and the product of the number of seed voxels and the number of samples per voxel; the final bidirectional connectivity index was then obtained as the mean of the two normalized directional values. Detailed tractography parameters are reported in Supplementary Table S4.

## 3. Results

### 3.1. VBM Results

Voxel-wise group comparisons of grey-matter volume were performed using a two-sample *t*-test within an a priori explicit mask encompassing the VF network, including DCI, PM, DLPFC, and MCC areas. To avoid potential bias, the following variables were included as covariates: site/scanner, age, full-scale IQ, and TIV.

**Figure 1:**
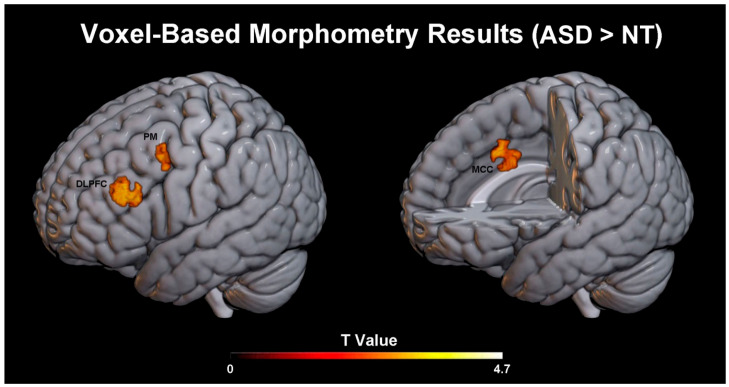
Voxel-based morphometry results within the VF network. Regions showing significantly greater grey-matter volume in ASD compared with NT controls are displayed (contrast ASD > NT, left hemisphere). Statistical inference is performed voxel-wise within an a priori explicit mask including the following ROIs: PM, DLPFC and MCC. Results are shown at cluster level (p_FWE < 0.05) and rendered on a standard MNI template.

Relative to the NT group, the ASD group showed significantly greater grey-matter volume in three regions within the VF network mask: PM, DLPFC, and MCC. As reported in Table 2, the most robust effect was observed in PM (T = 4.82, p_FWE = 0.008), while significant clusters were also detected in DLPFC (T = 4.56, p_FWE = 0.013) and MCC (T = 4.41, p_FWE = 0.017). Figure 1 illustrates the spatial distribution of these effects in the left hemisphere (for details see also Supplementary Figure 1). No significant group differences were detected in DCI. All reported effects survived family-wise error correction within the explicit VF network mask (voxel-wise p_FWE < 0.05; cluster-forming threshold *p* < 0.001, uncorrected). Notably, the reverse contrast (NT vs. ASD) did not yield any significant results.

### 3.2. TBSS Results

Voxel-wise TBSS analyses were performed within a priori defined white-matter ROIs associated with the VF ROIs and constrained to the FA skeleton (FA > 0.20). Group comparisons were assessed using permutation-based nonparametric testing (randomize) with TFCE and FWE correction (p_FWE < 0.05), controlling for site/scanner, age, full-scale IQ, and a diffusion motion index.

**Figure 2:**
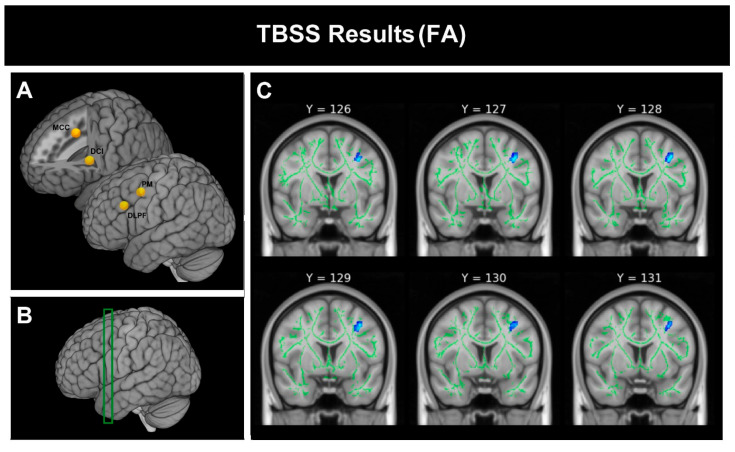
TBSS results for FA within the VF white-matter mask: (**A**) The panel shows the four ROIs that define the a priori VF network. (**B**) Location of the premotor-related white matter region shown in green color (**C**). (**C**) T-statistic map for the contrast NT > ASD indicating the significant cluster (blue color (p_FWE < 0.05).

Significant group differences were restricted to premotor-related white matter. As reported in Table 3, the NT group showed higher FA (97 skeleton voxels), higher AD/L1 (125 skeleton voxels), and higher F1 (112 skeleton voxels) than ASD within the PM white-matter ROI. In contrast, the ASD group showed higher RD in the same region (98 skeleton voxels). No significant group differences were detected in white-matter ROIs associated with DLPFC, MCC, or DCI. Figure 2 illustrates the spatial distribution of the FA effect within the premotor-related white matter. All effects survived TFCE-based family-wise error correction (p_FWE < 0.05).

### 3.3. Results of Correlation Analysis

Given that TBSS group differences in the white matter of the premotor area, correlation analyses were conducted for the ASD group to examine possible associations between premotor diffusion metrics and clinical severity measures. For each participant, mean diffusion values were extracted from the corresponding skeleton-constrained ROI. Associations with clinical severity measures were assessed using Spearman’s rank correlation. *p*-values were corrected using the Benjamini–Hochberg false discovery rate (FDR) due to the multiple comparisons performed.

Specifically, higher ADOS-2 Social Affect scores were associated with lower F1 values (ρ = −0.48, *p* = 0.03, q_FDR = 0.049); ADOS-2 calibrated severity scores were negatively correlated with F1 (ρ = −0.51, *p* = 0.02, q_FDR = 0.049) and with AD (L1) (ρ = −0.53, *p* = 0.01, q_FDR = 0.049). In addition, ADI-R Reciprocal Social Interaction scores showed a positive association with RD (ρ = 0.45, *p* = 0.04, q_FDR = 0.049) and a negative association with F1 (ρ = −0.46, *p* = 0.03, q_FDR = 0.049).

### 3.4. Results of Probabilistic Tractography

**Figure 3:**
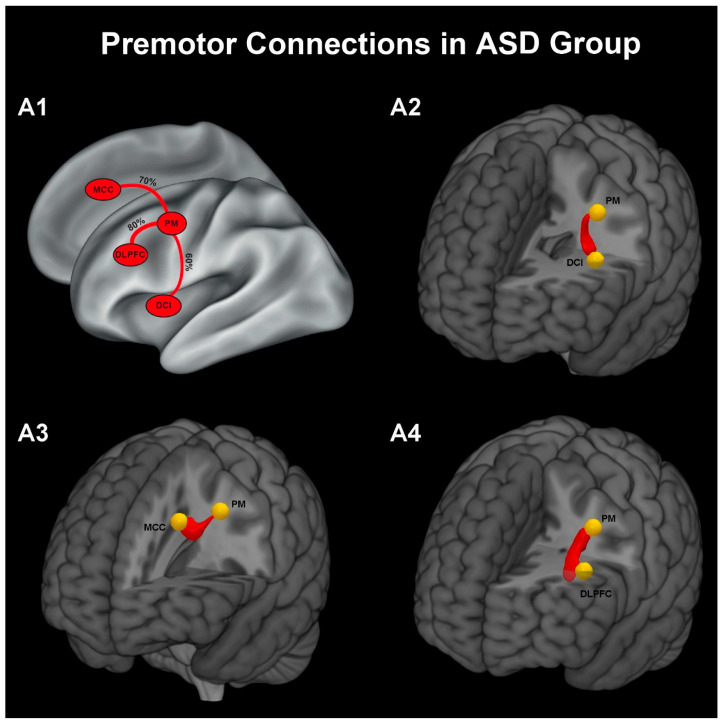
Overview of white matter pathways and their probability of connections with the PM in the ASD group: (**A1**). Three-dimensional reconstruction of averaged fibre tracts illustrating the pathway between PM and DCI (**A2**), PM and MCC (**A3**), PM and DLPFC (**A4**).

**Figure 4:**
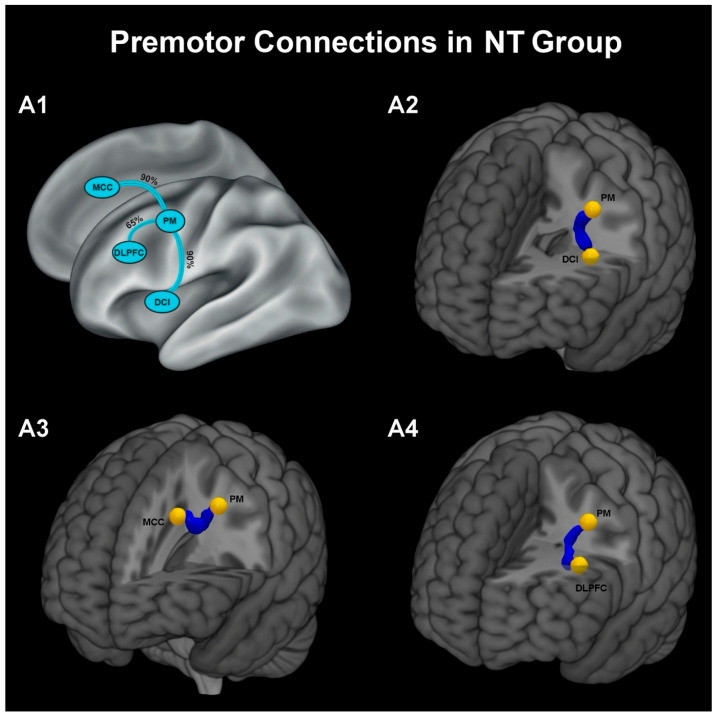
Overview of white matter pathways and their probability of connections with the PM area in the NT group: (**A1**). Three-dimensional reconstruction of averaged fibre tracts illustrating the pathway between PM and DCI (**A2**), PM and MCC (**A3**), PM and DLPFC (**A4**).

Probabilistic tractography was used to examine structural connectivity between the PM area and other nodes of the VF network. All three premotor-centred pathways, PM-DCI, PM-MCC, and PM–DLPFC, could be reconstructed in both groups, although with different reconstruction rates. In Figure 3 and Figure 4, panel A1 summarizes the reconstruction probability of each premotor-centred pathway, whereas panels A2–A4 illustrate the three-dimensional averaged tract reconstructions for the PM-DCI, PM-MCC, and PM-DLPFC connections, respectively. In the ASD group, the PM–DLPFC pathway was reconstructed in 80% of participants (16/20), whereas reconstruction rates were lower for the PM–MCC (70%, 14/20) and PM–DCI (60%, 12/20) pathways (Figure 3). In contrast, in the neurotypical group, reconstruction rates were highest for the PM–MCC and PM–DCI pathways (both 90%, 18/20), while the PM–DLPFC pathway was reconstructed in 65% of participants (13/20) (Figure 4). Between-group comparisons of tract reconstruction rates using Fisher’s exact test did not reveal statistically significant differences for PM–MCC (14/20 vs. 18/20, *p* = 0.235) or PM–DLPFC (16/20 vs. 13/20, *p* = 0.480). However, for the PM–DCI pathway, the lower reconstruction rate observed in the ASD group compared with the NT group (12/20 vs. 18/20) was close to the significant threshold (*p* = 0.06). Overall, the tract reconstruction patterns suggested a more consistent premotor connectivity with MCC and DCI regions in neurotypical children, whereas children with ASD showed a relatively greater prevalence of PM–DLPFC reconstructions. In line with this pattern, a quantitative analysis based on a connectivity index of tractography, for the PM–DCI pathway (considering the same covariates) also showed a trend-level group effect, with higher connectivity values in NT children than in the ASD group (F = 3.542, *p* = 0.058).

## 4. Discussion

The present study investigated possible structural and anatomical differences between ASD and NT peers within VFs, combining VBM, TBSS, and probabilistic tractography.

### 4.1. Grey-Matter Alterations Within Frontal and Cingulate Regions

The VBM results revealed increased grey-matter volume in children with ASD in the PM, DLPFC, and MCC. These findings are consistent with previous structural MRI studies reporting local cortical overgrowth of both cerebral grey and white matter in children with ASD, with the most severe enlargement occurring in frontal and cingulate cortices [32,33,34]. Additionally, this brain overgrowth may be due to an unusually rapid neural expansion occurring within the first growth phase of childhood (2–4 years), which is subsequently followed by abnormal slowing of growth and altered synaptic pruning processes [35]. This initial increase in cortical volume is later accompanied by atypical cortical thinning during subsequent developmental stages [32]. More recent studies employing predictive neuroimaging approaches have shown that early patterns of brain overgrowth, particularly involving cortical thickness, can predict an ASD diagnosis by 24 months of age [36]. Taken together, these findings support the hypothesis that an early excess of neural proliferation, followed by dysregulated pruning, may underlie the atypical cortical surface area and its connectivity typically observed in children with ASD. Importantly, these structural differences observed principally in brain areas such as the DLPFC and PM align with the functional role of these regions in VF processing. Indeed, the PM is crucial for encoding the kinematic and dynamic aspects of actions [37] and the DLPFC for higher-order control and contextual modulation of actions [38]. Regarding the PM area, these findings are also in line with monkey data showing that neurons of the premotor cortex encode various aspects of hand movement [38,39,40,41] including speed [42], distance [43] and reaction time for reaching [44,45]. In this view, an imbalance in grey-matter development across DLPFC, MCC, and especially PM, may therefore disrupt the fine-tuned integration required to translate affective states into action dynamics or to infer others’ affective mood/states from observed movements. However, VBM results also showed that children with ASD exhibit a cortical structure of DCI similar to that of NT children suggesting that impairments in VF encoding are not driven by local structural abnormalities of DCI, but rather by altered functional and/or structural connectivity between this region and other crucial nodes of the neural circuit such as DLPFC and PM. To address this issue, we next analyzed potential white-matter microstructural alterations using TBSS applied to diffusion DTI data.

### 4.2. Premotor White-Matter Microstructure and Clinical Severity

In all four ROIs (DCI, PM, DLPFC, MCC) TBSS was applied on DTI data of all children. This analysis revealed white-matter alterations only in the premotor cortex. Within this region, NT children showed higher FA, higher L1, and higher primary fibre volume fraction (F1), whereas children with ASD exhibited higher RD. Overall, this pattern suggests that NT children exhibit stronger and more organized white-matter pathways in the PM, whereas differences observed in ASD may reflect reduced fibre coherence, and/or altered axonal organization within premotor pathways. Importantly, within the ASD subgroup, premotor diffusion metrics were significantly associated with clinical severity measures. Greater social-affective impairment (ADOS-2 Social Affect and calibrated severity scores) was associated with lower F1 and L1 values, while ADI-R reciprocal social interaction scores were positively associated with RD. These associations suggest that reduced premotor microstructural integrity is related to core social-communicative symptomatology.

Notably, the correlation analyses of TBSS parameters computed in the PM region of the ASD group showed that diffusion metrics were significantly associated with clinical severity measures. Greater social-affective impairment (ADOS-2 Social Affect and calibrated severity scores) was associated with lower F1 and L1 values, while ADI-R Reciprocal Social Interaction scores were positively associated with RD. These associations suggest that reduced microstructural integrity observed in the premotor area is related to core social-communicative symptomatology. Although causality cannot be inferred, the convergence between group differences and symptom correlations strengthens the interpretation that premotor white-matter organization is clinically relevant in ASD. These findings are consistent with previous research suggesting that structural alterations in specific morphological brain features may be associated with emotional difficulties and autism severity [46]. As proposed by Di Cesare et al. [5], the PM may be involved in transforming affective information received from the DCI into motor output. In this context, alterations in white-matter organization within this region could potentially contribute to atypical perception [5,7,47] as well as differences in the expression [9,48] of VF observed in children with ASD. Reduced efficiency in the connections between the DCI and premotor regions might limit the encoding of affective information related to VFs during both observation (from DCI to PM) and execution (from PM to DCI). Nevertheless, further research is needed to clarify the nature of the relationship between the neural structure of the VF network and difficulties in VF expression and recognition.

### 4.3. Network-Level Connectivity Patterns

To study the anatomical connectivity of PM with the other three ROIs (DCI, MCC, DLPFC), probabilistic tractography was applied to DTI data. While all three PM-centred tracts (PM–DCI, PM–MCC, PM–DLPFC) could be reconstructed in both groups, their connection rates differed systematically. Indeed, in children with ASD, connectivity regarding the PM-DCI tract (ASD: 60%; NT: 90%) and PM-MCC tract (ASD: 70%; NT: 90%) was less consistent, whereas the PM–DLPFC tract showed a relatively higher connectivity rate compared to NT children (ASD: 80%; NT: 65%). While these findings are descriptive rather than inferential, they suggest a potential shift in network organization, with reduced consistency of premotor–insula connectivity and relatively preserved or enhanced premotor–prefrontal coupling in ASD. One possible interpretation of this network connectivity suggests that affective-motor integration may rely more strongly on prefrontal control mechanisms in ASD, rather than on more automatic premotor–insula interactions typically observed in neurotypical development. This interpretation is consistent with behavioural evidence indicating greater reliance on explicit or cognitively mediated strategies during social processing in ASD [8].

## 5. Limitations and Future Directions

Some limitations should be acknowledged. First, diffusion MRI data were available only for a subset of participants, reducing statistical power and limiting the generalisability of microstructural and tractography findings. Second, the multisite nature of the ABIDE dataset introduces heterogeneity in acquisition parameters; although site/scanner and a diffusion motion index were modelled as nuisance covariates, residual site- and motion-related confounds cannot be fully excluded. Third, the sample included only right-handed male children with a full-scale IQ > 70 and was restricted to an age range of 6–10 years, limiting the extension of the findings to females, individuals with intellectual disability, and other developmental stages. Fourth, Vitality Form-specific behavioural measures were not available in this imaging cohort, preventing direct brain–behaviour inferences about VF perception and expression. Finally, tractography results were primarily reported as reconstruction rates and group overlap visualizations; future work should complement these descriptive indices with quantitative tract-level metrics and confirm findings in independent cohorts. Future studies should integrate neuroanatomical measures with task-based behavioural assessments of VF perception and production within the same participants, enabling more direct tests of the proposed neural mechanisms. Extending analyses across broader developmental windows, including females and more heterogeneous ASD presentations, will be essential to clarify developmental trajectories and generalisability.

## 6. Conclusions

In conclusion, this study highlights three main findings. First, children with ASD exhibited increased grey-matter volume in the PM, DLPFC, and MCC. Second, the white-matter microstructural organization of the PM differed in children with ASD compared with NT children. Third, analysis of anatomical connectivity among the ROIs revealed reduced connectivity between the PM and the DCI, alongside increased connectivity between the DLPFC and the PM in ASD. Together, these alterations may underlie core difficulties in both the expression and interpretation of action Vitality Forms. By identifying the premotor cortex as a key hub linking motor execution with affective communication, these findings support a more embodied account of social deficits in autism and open new avenues for targeted interventions focusing on action VFs and motor–affective integration.

## Figures and Tables

**Table 1:** Clinical characteristics of the sample. Values are reported as mean ± standard deviation unless otherwise specified; site distribution is reported as n (%). T1-weighted MRI data are available for 30 children per group, while diffusion MRI data are available for a subset of 20 children per group. ADOS-2 and ADI-R scores are reported for the ASD group only.

	ASD Children		NT Children	
	T1 Sample (n = 30)	dMRI Subset (n = 20)	T1 Sample (n = 30)	dMRI Subset (n = 20)
Age (years)	8.74 ± 1.58	8.82 ± 1.47	8.50 ± 1.26	8.76 ± 1.15
Sex	Male	Male	Male	Male
Full-scale IQ	103 ± 19	103 ± 18	118 ± 14	119 ± 14
NYU	57%	45%	60%	55%
SDU	30%	40%	27%	35%
Trinity	13%	15%	13%	10%
ADOS-2 SA	11.35 ± 4.12	11.82 ± 4.53	n.a.	n.a.
ADOS-2 Severity	8.08 ± 1.74	8.12 ± 1.90	n.a.	n.a.
ADOS-2 Total	15.27 ± 5.01	15.60 ± 5.44	n.a.	n.a.
ADI-R RSI	17.43 ± 5.62	17.15 ± 5.53	n.a.	n.a.

**Table 2:** Coordinates and statistical values of the VBM analysis revealing significantly increased grey matter volume in the ASD group relative to NT.

Region	Peak MNI (x y z)	kE (Voxels)	T	p_FWE
PM	−38 4 45	208	4.82	0.008
DLPFC	−48 33 26	390	4.56	0.013
MCC	−3 15 40	390	4.41	0.017

**Table 3:** TBSS results within premotor-related white matter. Significant skeleton voxels (p_FWE < 0.05) are reported for each diffusion metric and contrast direction.

Metric	Region	Direction	#Skeleton Voxels	p_FWE (TFCE)
FA	PM WM	NT > ASD	97	<0.05
AD (L1)	PM WM	NT > ASD	125	<0.05
F1	PM WM	NT > ASD	112	<0.05
RD	PM WM	ASD > NT	98	<0.05

## Data Availability

The data were obtained from the Autism Brain Imaging Data Exchange (ABIDE) public database (http://fcon_1000.projects.nitrc.org/indi/abide/, accessed on 19 April 2026).

## Supplementary Materials

The following supporting information can be downloaded at: https://www.mdpi.com/article/10.3390/brainsci16050446/s1, Table S1. T1-weighted structural MRI acquisition parameters (site-specific). Table S2. Diffusion MRI (DTI) acquisition parameters (site-specific). Table S3. Peak coordinates used to define the a priori VFs network explicit mask (spherical ROIs in MNI space; radius = 5 mm). Table S4. Probabilistic tractography parameters.
