# Supplementary material for: Structural and Connectivity Alterations of the Premotor Cortex in Autistic Children: Implications for Affective Motor Impairments
Source: Brain Sci. 2026 Apr 23;16(5):446. doi: 10.3390/brainsci16050446 (PMC13204433; doi:10.3390/brainsci16050446)
Supplement: Supplementary file 1 [file brainsci-16-00446-s001.zip › brainsci-4219898-supplementary.pdf]

Supplementary

Table S1. T1-weighted structural MRI acquisition parameters (site-specific)

Site	Scanner	Coil	Sequence	TR (ms)	TE (ms)	TI (ms)	Flip (°)	Matrix	FOV (mm)	Voxel (mm)	Slices	Acq time (min:s)
TCD	Philips Achieva 3T	8Ch	MPRAGE	8.4	3.9	1150	8	256×256	230×230	0.9×0.9×0.9	190	5:43
NYU	Siemens Allegra 3T	8Ch	3D TFL	2530*	3.25	1100	7	256×192	256×256	1.3×1.0×1.33	128	8:07
SDSU	GE MR750 3T	8Ch	3D SPGR (FSPGR)	8.136	3.172	600	8	256×192	256×256	1.0×1.0×1.0	172	4:54

Table S2. Diffusion MRI (DTI) acquisition parameters (site-specific)

Site	Scanner	Sequence	TR (ms)	TE (ms)	Voxel (mm)	Slices	Directions	b-value(s) (s/mm²)	#b0	Fat suppr.	Acq time (min:s)	Fieldmap corr.
TCD	Philips Achieva 3T	EPI (DTI)	20244	79	1.94×1.94×2.0	65	61	1500	4	SPIR	24:21	not specified
NYU	Siemens Allegra 3T	EPI (DTI)	5200	78	3.0×3.0×3.0	50	64	1000	1	None	5:43	Field map acquired
SDSU	GE MR750 3T	EPI (DTI)	8500	84.9	1.875×1.875×2.0	68	61	1000	1	Yes	-	Fieldmap corrected

Table S3. Peak coordinates used to define the a priori VFs network explicit mask (spherical ROIs in MNI space; radius = 5 mm).

Region	Hemisphere	MNI x	MNI y	MNI z	Radius (mm)	Source
Dorso-Central insula (DCI)	L	-38	10	-4	5	Di Cesare et al. 2025
Premotor Cortex (PM)	L	-46	4	44	5	Di Cesare et al. 2025
Middle Cingulate Cortex (MCC)	L	-6	10	38	5	Di Cesare et al. 2021
Dorsolateral Prefrontal Cortex (DLPFC)	L	-42	32	32	5	Di Cesare et al. 2025

Table S4. Probabilistic tractography parameters

Parameter	Value
Samples per seed voxel (P	10000
Maximum number of steps (S)	1000
Step length	0.5 mm
Curvature threshold (c)	0.3
Waypoint condition	AND
Tracking direction	Bidirectional (A→B and B→A)
Tracking space	Native diffusion space
Fibre model	bedpostx (two fibres per voxel)
Tracking mask	Subject-specific brain mask

Methods: Probabilistic tractography

ROIs were manually defined in native diffusion space for each participant. For each anatomical region (PM, insula, DLPFC), an expert operator identified a single seed voxel, which was expanded into a spherical ROI with a 5-mm radius using morphological dilation. All ROIs were binarised and used as seed and target masks for tractography. Diffusion data were modelled using a two-fibre Bayesian crossing-fibre model implemented in bedpostx. Probabilistic tractography was performed using probtrackx2 with waypoint-constrained tracking.
